# The Role of Complementary and Alternative Medicine in Regenerative Medicine

**DOI:** 10.1155/2013/420458

**Published:** 2013-09-19

**Authors:** Yueh-Sheng Chen, Wei-Chiang Lin, Cheryl Miller

**Affiliations:** ^1^School of Chinese Medicine, China Medical University, Taichung 40402, Taiwan; ^2^Department of Biomedical Engineering, Florida International University, Miami, FL 33174, USA; ^3^Department of Surgery, Saint Louis University, St. Louis, MO 63103, USA

Regenerative medicine is the process of replacing or regenerating animal cells, tissues, or organs to restore or establish normal function. This field has the potential to solve the problem of the shortage of organs available for donation and of organ transplant rejection. Recently, application of Complementary and Alternative Medicine (CAM) as a means to accelerate the process of regeneration is a new approach. The CAM therapies offer a natural and cost-effective intervention to change the course of chronic disease and may regenerate failing organ systems.

 The paper by Dr. Y.–J. Chen et al. explores how the Hepatitis B virus-encoded X regulates the expression of epidermal growth factor receptor, an important gene for growth of hepatocytes. Dr. H.–Y. Liu et al. demonstrated the regenerative potentials of deep sea water on osteogenesis, showing that deep sea water could potentially be applied in osteoporosis therapy. Dr. S. –H. Hsu et al. prepared peripheral nerve conduits containing the negatively charged *Tremella fuciformis* polysaccharide (TF) and successfully used the TF-immobilized conduits to repair a large (15 mm) critical gap defect in rats. Y. Lee et al. found that L-glutamate-induced neurotoxicity could be suppressed by the treatment with constituents of *Rhodiola rosea*, indicating that the *Rhodiola rosea* may have therapeutic potential for the treatment of inflammation and neurodegenerative disease. Dr. B.–Y. Yang et al. tried to apply percutaneous electrical stimulation to improve bone remodeling and bone healing in rats. X-ray and micro-CT showed that the electrical treatment could increase the amount of newly formed cranial bone. W. Liu et al. examined the contribution of side population (SP) cells from kidney and bone marrow to the reconstitution of kidney SP pools after ischemia-reperfusion injury (IRI). They found that following renal IRI, kidney SP cells were acutely depleted and then progressively restorated to baseline levels by both self-proliferation and extrarenal source, that is, bone marrow-derived cell homing. Dr. F. Yahya et al. showed that methanol extract of *Bauhinia purpurea* leaves could exert potential hepatoprotective activity in rats via its antioxidant activity and high phenolic content. T. Jayakumar et al. reviewed the effects of andrographolide, a major bioactive chemical constituent in *Andrographis paniculata* (Burm.f.) Nees, against cardiovascular disease, platelet activation, infertility, and NF-kB activation. Dr. H.–M. Chiang et al. showed the antioxidant activity of a *Neonauclea reticulata* water extract against ultraviolet B (UVB) irradiation in human skin fibroblast cell cultures (Hs68) by inhibiting MMP-1, -3, and -9 expressions and increasing levels of collagen activity. Dr. W.–Y. Su et al. developed a biphasic calcium phosphate cement, consisting of *α*-tricalcium phosphate (*α*-TCP) and hydroxyapatite (HAP), which is a potential biomaterial for bone repair. C. Y. Ho et al. showed that acupuncture and electroacupuncture could have positive effects on regeneration of median nerve in rats. J. Xiao et al. demonstrated that administration of garlic-derived antioxidant S-allylmercaptocysteine (SAMC) could ameliorate hepatic injury in a nonalcoholic fatty liver disease rat model. Dr. S.–C. Lee et al. investigated the effect of ferulic acid (FA) against peripheral nerve injury. They found that FA appears to promote peripheral nerve regeneration across a 15 mm critical defect gap in the rat sciatic nerve injury model. Suppression of macrophages by FA at the site of peripheral nerve injury may contribute to its nerve growth-promoting capability.

By compiling these articles, we hope to stimulate our readers and researchers to provide continuing efforts to fully understand the effects of CAM therapies on regenerating tissues or organs.

*Yueh-Sheng Chen* *Yueh-Sheng Chen* *Wei-Chiang Lin* *Wei-Chiang Lin* *Cheryl Miller* *Cheryl Miller*

